# Proteome-Wide Analysis of Single-Nucleotide Variations in the N-Glycosylation Sequon of Human Genes

**DOI:** 10.1371/journal.pone.0036212

**Published:** 2012-05-07

**Authors:** Raja Mazumder, Krishna Sudeep Morampudi, Mona Motwani, Sona Vasudevan, Radoslav Goldman

**Affiliations:** 1 Department of Biochemistry and Molecular Biology, George Washington University Medical Center, Washington, D.C., United States of America; 2 Department of Oncology, Georgetown University, Washington, D.C., United States of America; 3 Department of Biochemistry and Molecular and Cellular Biology, Georgetown University Medical Center, Washington, D.C., United States of America; University of Westminster, United Kingdom

## Abstract

N-linked glycosylation is one of the most frequent post-translational modifications of proteins with a profound impact on their biological function. Besides other functions, N-linked glycosylation assists in protein folding, determines protein orientation at the cell surface, or protects proteins from proteases. The N-linked glycans attach to asparagines in the sequence context Asn-X-Ser/Thr, where X is any amino acid except proline. Any variation (e.g. non-synonymous single nucleotide polymorphism or mutation) that abolishes the N-glycosylation sequence motif will lead to the loss of a glycosylation site. On the other hand, variations causing a substitution that creates a new N-glycosylation sequence motif can result in the gain of glycosylation. Although the general importance of glycosylation is well known and acknowledged, the effect of variation on the actual glycoproteome of an organism is still mostly unknown. In this study, we focus on a comprehensive analysis of non-synonymous single nucleotide variations (nsSNV) that lead to either loss or gain of the N-glycosylation motif. We find that 1091 proteins have modified N-glycosylation sequons due to nsSNVs in the genome. Based on analysis of proteins that have a solved 3D structure at the site of variation, we find that 48% of the variations that lead to changes in glycosylation sites occur at the loop and bend regions of the proteins. Pathway and function enrichment analysis show that a significant number of proteins that gained or lost the glycosylation motif are involved in kinase activity, immune response, and blood coagulation. A structure-function analysis of a blood coagulation protein, antithrombin III and a protease, cathepsin D, showcases how a comprehensive study followed by structural analysis can help better understand the functional impact of the nsSNVs.

## Introduction

Protein glycosylation, one of the most common post translational modifications, involves enzymatic addition of oligosaccharides, also referred to as glycans or carbohydrates, to proteins. The oligosaccharides commonly found on a protein are either N-linked or O-linked. N-linked glycans, the focus of this paper, are attached to the –NH2 group of asparagines. N-glycosylation of proteins strongly influences their function including folding, cellular localization, and turnover [1]–[7]. A specific NX(S/T) amino acid sequence motif is commonly required for N-glycosylation of proteins. In this sequon, N is asparagine, S/T is either serine or threonine, and X is any amino acid except proline [5]. Not all NX(S/T) sequences in protein molecules are glycosylated because some are not accessible to the glycosylation enzymes [8]. Other sequence motifs for the attachment of N-glycans have been described but are utilized with much lower frequency [9].

**Figure 1:**
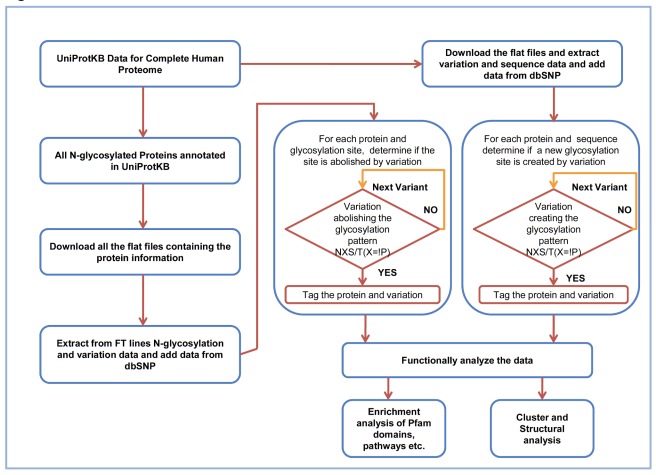
Flow chart of the method used to identify polymorphic glycoproteins.

Although the general importance of N-glycosylation is acknowledged, the actual N-glycoproteome of an organism is only beginning to emerge [9]. Decoding the function of glycoproteins in a comprehensive manner is one of the major challenges of the post-genomic era. Because changes in the structure of N-glycans attached to the glycoproteins are sufficient to cause disease, research on N-glycosylation in the disease context is particularly important [3]. Mutations in glycosyltransferases, leading to truncated N-glycans, cause congenital disorders of glycosylation [10]. More subtle examples include highly branched sialylated N-glycoproteins that facilitate metastatic cancer diseases [2] [11]. The protein N-glycosylation signals are further amplified by an entire glycan recognition network including galectins, siglecs and other interacting partners which magnify their functional impact [12]. The evidence for causality in such complex phenotypes may be difficult to establish [13]. However, complex changes in the N-glycan structures are not the only possible modification; direct evidence also links variations in the NX(S/T) sites on individual proteins to diseases [14]–[18].

**Table 1:** Summary of results of loss and gain of N-glycosylation in the human proteome.

Description	Number
Human proteins annotated as N-glycosylated in UniProtKB/Swiss prot	3680
Confirmed N-linked glycoproteins	967
Proteins that have one or more glycosylation sites abolished due to nsSNV	226
Glycosylation sites lost due to nsSNV	259
Proteins that have one or more new glycosylation sites formed due to nsSNV	917
Glycosylation sites gained due to nsSNV	1091
Protein variants that have structural information at the N-glycosylation site (includes loss and gain sites)	279

**Table 2:** Cellular component that the protein occupies as defined in UniProtKB/Swiss-Prot keyword^1^.

Cellular component	# of proteins in complete proteome^2^	# of nsSNV carrying glycoproteins^3^
Secreted	1814	178
Membrane	6714	477
Cytoplasm	3987	204
Nucleus	4690	218

1 The same proteins can have more than one keyword if it is found to be present in more than one location.

2 15293 out of the total 20242 proteins in the human proteome have a Cellular Component keyword.

3 935 out 1091 nsSNV glycoproteins have a Cellular Component keyword.

According to a recent study, 27 out of 31 mutations of the NX(S/T) motif were found to be associated with genetic diseases [19]. Mutations leading to the gain of glycosylation have been associated with myoclonus-dystonia syndrome [20]; growth retardation due to inhibited secretion of insulin-like growth factor-binding protein [21]; and rolandic seizures [22]. Susceptibility to mycobacterial infections was shown to be caused by the T168N gain of N-glycosylation mutantation of IFNGR2 [14]. Kretz et al. showed that abolition of a glycosite in saposine B results in accelerated proteolysis and metachromatic leukodystrophy. The authors identified several other human proteins affected by the proximity of a proteolytic site to the N-glycosylation sequon [23]. Because the N-glycosylation sites have been shown to be protected and selection mechanisms that fix the N-glycosites in the population have been suggested [24], it is plausible that SNVs affecting N-glycosylation sites contribute significantly to human diseases [25]. For example, the D327N variant of the sex hormone-binding globulin (SHBG) which creates a new glycosylation site was shown to be protective in breast cancer [26], [27]. It was suggested that distribution of SHBG between circulation and tissues is affected by the N-glycosylation status at the D327N polymorphic site [28]. This underscores the need for further research on the impact of the N-glyco variants on protein function and human diseases.

**Table 3:** Frequency of amino acids that are part of the glycosylation motif.

Amino acid	# in completeproteome	# in gly motif	# of nsSNV that abolishes/creates anamino acid that is located outside of N-gly	# of nsSNV that results in loss/gain of N-gly
N	438455	18662	4188/3990	126/553
S	991278	10268	8390/8867	54/303
T	617501	8394	7265/7258	90/243

Our goal is to build a framework for better understanding the effects of non-synonymous single nucleotide variations (nsSNV) on the N-glycosylation of proteins. We focus on a comprehensive analysis of the non-synonymous (missense) substitutions that lead to either loss or gain of the N-glycosylation motif. As described above, there are examples of the impact of individual polymorphic sites but, surprisingly, a comprehensive survey of the impact of SNV on the N-glycosylation sequon has not been presented to the best of our knowledge. To assess the overall impact of the nsSNV on the human proteome, we have extracted the N-glycosylation and variation data from the UniProtKB/Swiss-Prot [29] and dbSNP data from PolyPhen [30]. The survey is followed by evaluation of functional enrichment within our dataset with specific examples of protein structure-function analysis. We expect that this compact information and novel use of proteome-wide data will provide researchers with improved tools for the design of innovative studies to understand the impact of nsSNV on human diseases.

**Figure 2:**
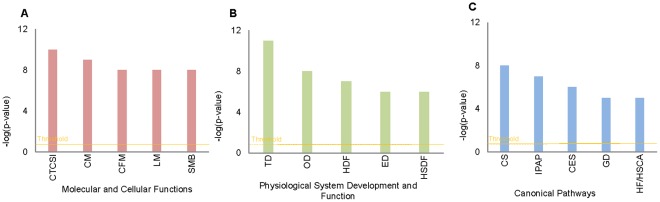
IPA results for functional analysis of the protein data set. The threshold for all the analyses was set to p<0.05 and the data is plotted against –log of p values. The top three categories with lowest p value are presented for three IPA categories: **A**. molecular and cellular functions; CTCSI -Cell to Cell Signaling and Interaction, CM- Cellular Movement, CFM- Cellular Function and Maintenance. **B**. physiological system development and functions; TD- Tissue Development, OD- Organismal Development HSDF- Hematological System Development and Function. **C**. canonical pathways; CS- Coagulation System, IPAP- Intrinsic Prothrombin Activation Pathway, CES- Caveolar mediated Endocytosis Signaling.

## Materials and Methods

The complete human proteome was obtained from the manually curated UniProtKB/Swiss-Prot (UniProt release 2011_01) [29]. The nsSNV variation data were extracted from UniProtKB/Swiss-Prot and PolyPhen (accessed July 2010) [30] which provides dbSNP data mapped to UniProtKB/Swiss-Prot. All proteins that lost or gained one or more NX(S/T) glycosylation motif/s due to missense genomic variation were recorded for further annotation and analysis. Information on pathways, structure, Pfam domain and disease association was collected from UniProtKB. All proteins that gained glycosylation sites were evaluated using the N-glycosylation prediction tool NetNGlyc [http://www.cbs.dtu.dk/services/NetNGlyc/]. Prediction of the N-terminal targeting sequences for endoplasmic reticulum was performed using Predotar [31].

**Figure 3:**
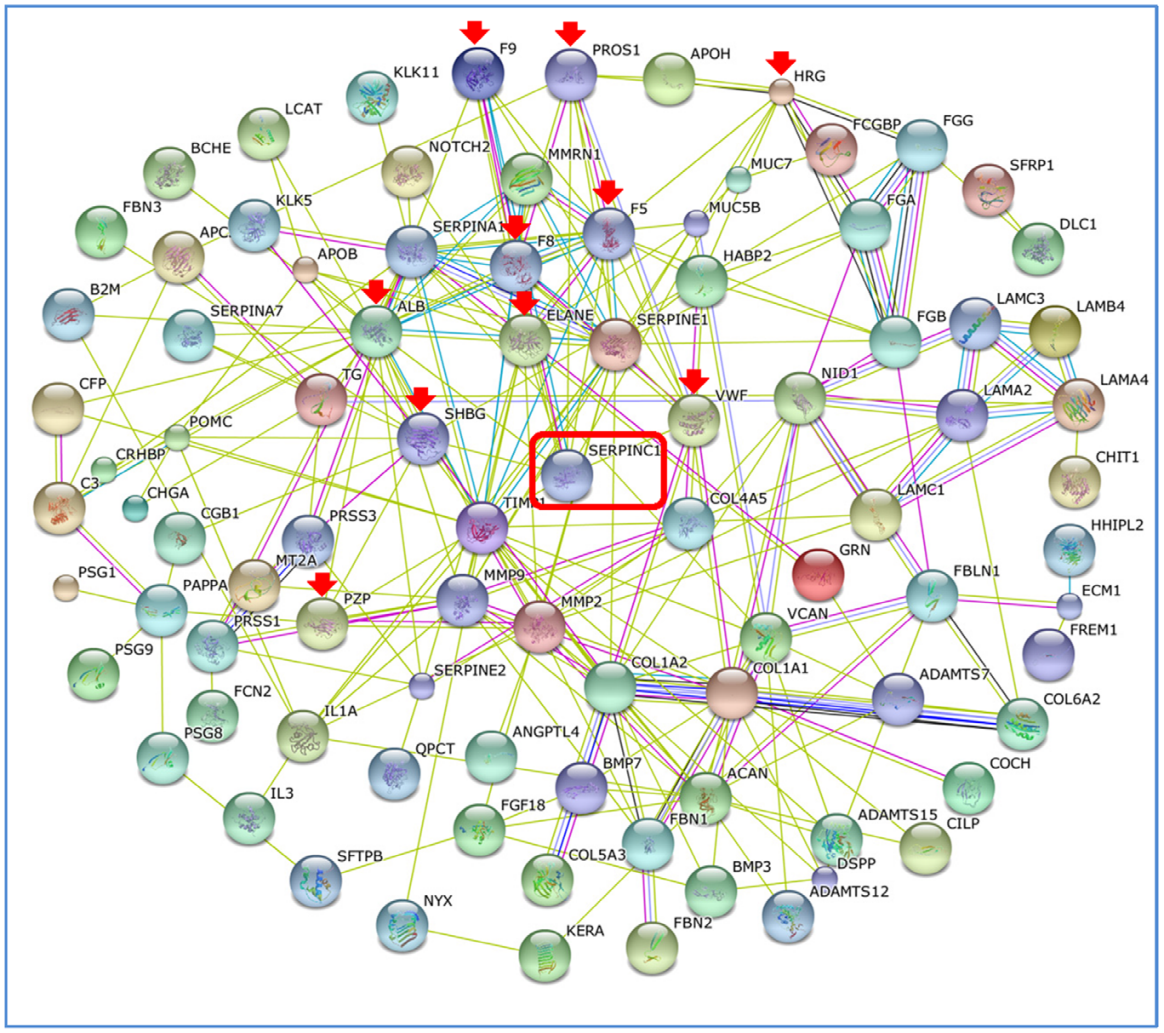
Network of the interactions of secreted proteins. Multiplicity of lines connecting individual proteins indicates the level of evidence for the interactions. A green line - neighborhood evidence; a blue line - concurrence evidence; a purple line - experimental evidence; a light blue line - database evidence; a black line – co-expression evidence. Antithrombin III (SERPINC1) is boxed in red and the interacting partners are marked with red arrows.

Enrichment analysis was performed to identify over- or under- represented Pfam domains in the loss and gain of glycosylation dataset, compared to their occurrence in the entire human proteome. The expected occurrence of a domain in the dataset was calculated based on the actual number of times it is present in the human proteome. A p value of 0.05 was considered significant and was calculated based on methodology described earlier [32]. In order to understand the nature of variations in the protein families, all the proteins in the data set were clustered using iProClust [http://pir.georgetown.edu/cgi-bin/iProClust.pl]. This process iteratively clusters the closely related proteins based on their length and similarity. Pathways were analyzed using Ingenuity Pathway Analysis® (IPA) tools, a web based interface for Ingenuity Knowledge Base (Ingenuity® Systems, http://www.ingenuity.com).

**Figure 4:**
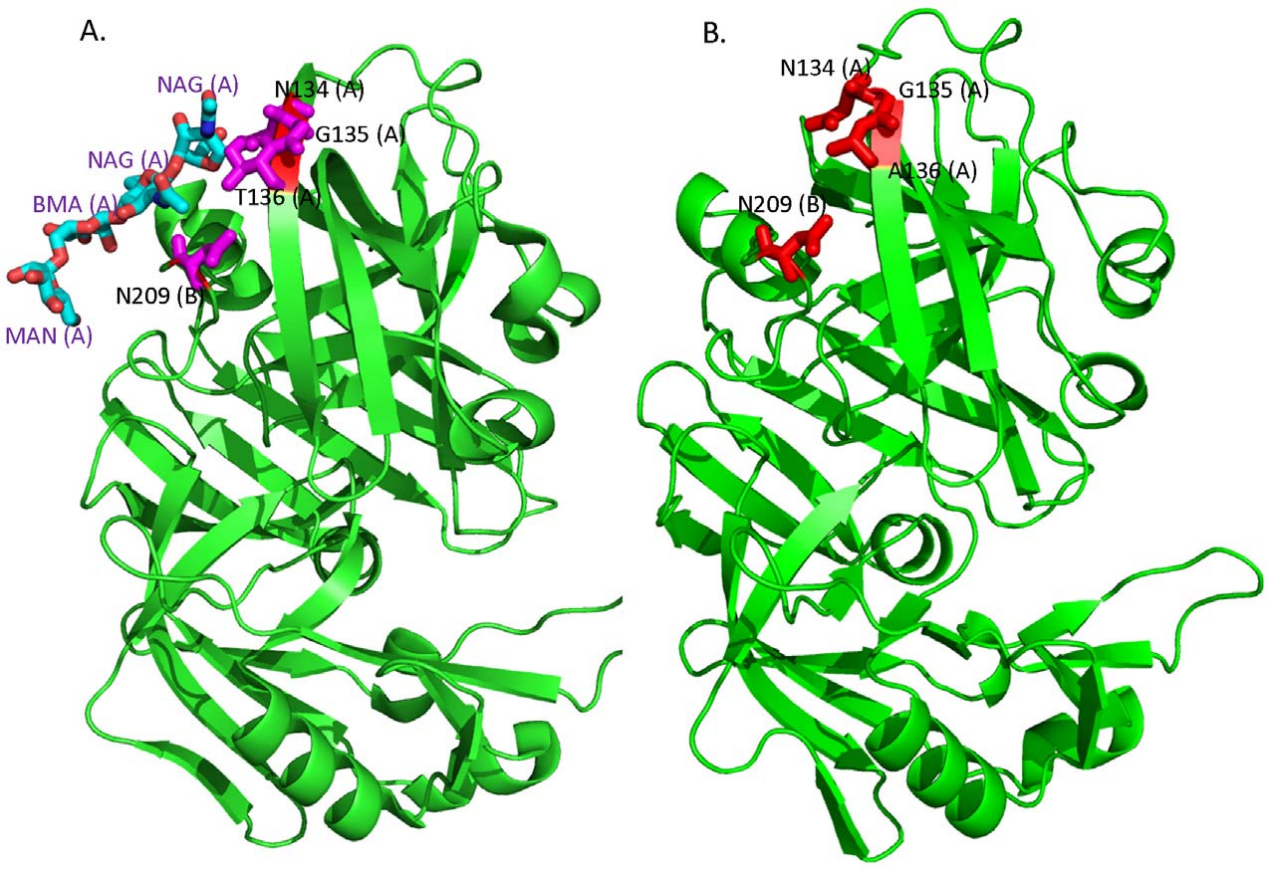
Effect of loss of N-linked glycosylation in cathepsin D due to nsSNV. **A**. Crystal structure of cathepsin D (PDB id: 1LYA) in complex with a bound N-linked oligosaccharide. The glycosylation motif extends from a loop to a strand with the N in the loop and the remainder in the strand. The interacting residues in pink from Chain A (134NGT136) and N209 from Chain B are shown as sticks. Oxygen atoms are colored in red and nitrogens in blue. The oligosaccharide (NAG-NAG-BMA-MAN) forms a hydrogen bond with N134, T136 and N209. **B**. Crystal structure of cathepsin D (PDB id: 1LYA) depicting the variation at position 136 from T to an A. There is a change in the charge distribution resulting in a loss of a hydrogen bond. This may result in the loss of the glycosylation site. A and B within parenthesis represent the chain Ids within the PDB.

To calculate the representation of nsSNVs within the human proteome or a dataset, the calculations were done in the following manner: total number of nsSNVs in human proteome divided by length of proteome (sum of the protein lengths); total number of nsSNVs in dataset divided by the length of all the proteins in the dataset. The p value was calculated based on methodology described earlier [32].

PyMOL (www.pymol.org) was used to model the amino acid variation and generate the structure figures. The accessible solvent area was checked by ASAView [33]. The homology model was built by using the SWISS_MODEL [http://swissmodel.expasy.org/] and the structural comparison was done using FATCAT [http://fatcat.ljcrf.edu/fatcat-cgi/cgi/fatcat.pl?-func=pairwise].

## Results and Discussion

The human proteome available from UniProtKB/Swiss-Prot contains curated information in the feature (FT) lines with regards to experimentally characterized and predicted glycosylation sites [29]. The database entries also provide information on nsSNV. The dbSNP database at NCBI [34], [35] also has information on missense variations. A comparison of dbSNP with UniProtKB/Swiss-Prot shows that, although there is some overlap, there is variation data unique to just one of the databases. Therefore, a comprehensive analysis of the impact of sequence polymorphism on the loss/gain of protein glycosites requires merging of the information from dbSNP and UniProt. In this study, we focus on the analysis of non-synonymous substitutions that lead to either loss or gain of the N-glycosylation motif. All the variations were mapped for further analysis to the reference human proteome available from UniProtKB/Swiss-Prot.

### Impact of nsSNV on the NXS/T Motif

To identify proteins that lose the NXS/T glycosylation sequon due to nsSNV, all the glycosylated proteins from the human proteome were extracted from UniProtKB. A flowchart describing the process is presented in Figure 1 and a summary of these findings is presented in Table 1 (the entire dataset is available in Table S1). UniProtKB/Swiss-Prot annotates 4421 proteins (22% of human proteins) with N- and O- linked glycosylation sites. Of these, 3680 proteins (18% of the human proteome) have one or more N-linked glycosylation sites. This is a fraction of the 15,313 proteins with NXS/T (where X is not a P) motifs in the human proteome, however, there is evidence that only fraction a of the existing sites are in fact glycosylated [9]. The information on occupancy of N-glycosylation sites in human is at the moment incomplete but the most complete annotation of glycosylated proteins is likely the UniProtKB/Swiss-Prot database. For identifying proteins that lose the glycosylation motif, we therefore considered in our analysis the 3680 proteins annotated as N-glycosylated by the UniProtKB/Swiss-Prot which is considered the gold standard set of manually curated human proteins.

On mapping the variation data to the UniProtKB/Swiss-Prot human proteome, we find that 259 unique variations resulted in the loss of N-glycosylation in 226 proteins (6% of the proteins that are N-glycosylated). A total of 28 proteins are predicted to contain more than one NXS/T site lost due to the nsSNV (see Table 1 and Table S2). Similarly, the proteins that gain the N-glycosylation motif due to nsSNV were identified by mapping the variation data from UniProtKB and PolyPhen to the UniProtKB/Swiss-Prot human proteome. It was found that 1073 unique variations resulted in gain of the N-glycosylation motif in 917 proteins (5% of the human proteome). In total, 1091 proteins (loss plus gain) were found to have variation at the N-glycosylation sequon. 113 proteins gained more than one NXS/T site due to the nsSNV (see Table 1 and Table S2). Even though the likelihood of having two nsSNVs affecting the protein in the same person is low, it is important to note that the number of ways the protein can gain new N-glycosylation site increases. The majority of the proteins in our dataset gained a single new N-glycosylation site due to nsSNV; some of the non-glycoproteins acquired one or more NXS/T motifs due to variation. Of 563 non-glycoproteins, 524 gained one and 39 gain 2–4 new NXS/T sites, respectively (Table S2). In the loss set, 19 proteins lost the only existing N-glycosylation site and an additional 5 proteins with two N-glycosylation sites had both sites modified by nsSNV. The conversion of a protein from non- to N- glycosylated is the most dramatic change but the changes in N-glycosylation multiplicity cannot be disregarded in view of results demonstrating that the total number of N-glycan branches affects function [24].

Based on UniProtKB/Swiss-Prot annotation of the 1091 proteins, 178 proteins are annotated with the keyword “secreted” and 477 with keyword “membrane” (Table 2). Our data set also contains many cytoplasmic and nuclear proteins which most likely are not glycosylated [9]. However, the information on localization of the proteins and their N-glycosylation in human population is incomplete and in many cases dependent on the state of the cell. For example, UniProtKB/Swiss-Prot annotates 65 out of the 204 cytoplasmic proteins with a new NXS/T (Table 2) also as either membrane bound or secreted. Similarly, out of 218 nuclear proteins, 36 are annotated as membrane bound or secreted at some point of their life cycle. In addition, the location of proteins may change in a disease context which may not be currently captured in UniProtKB/Swiss-Prot annotation. Further analysis of the proteins that gain glycosylation motifs by the NetNGlyc server shows that 84% of the proteins with new NXS/T sequon are predicted to be glycosylated using default cutoffs (data not shown). This high percent of positive predictions indicates that the SNVs might indeed have a functional impact, though it is possible that some of them could be false positives (there are also false negatives; for example SHBG is not predicted to be glycosylated by the NetNGlyc server but is known to be glycosylated [27]). To further check the results obtained from NetGlyc we used the neural-network-based prediction program Predotar which is capable of identifying endoplasmic reticulum (ER) signal peptides (ER is believed to be the site of addition of N-linked oligosaccharides). Out of the 1091 proteins that were identified to be affected by nsSNVs, 37% pass through the endoplasmic reticulum and 56% were predicted to be “mitochondrial” or had no targeting sequence (Table S3). We analyzed these proteins and found that, even though they did not have ER targeting sequences, several of them have been experimentally proven to be N-glycosylated. Examples of such proteins include glucosylceramidase (GLCM_HUMAN) [36], [37], antithrombin III (ANT_HUMAN), [38]–[40], sphingomyelin phosphodiesterase (ASM_HUMAN) [41] and many others. Based on our evaluation of the NetNGlyc and Predotar’s results, we conclude that the entire dataset, irrespective of the prediction results, should be functionally analyzed to avoid any bias that might be present in the prediction programs and current annotation in the databases. To stimulate experimental verification of the gain and loss of glycosylation sites, the entire dataset with Predotar predictions are provided in Table S3.

Recent work by Park and Zhang comparing mouse, rat, chicken, and human N-glycosylation shows that the NXS/T motifs that contain glycosylated asparagines are more conserved (in a panel of organisms) than the motifs that have non-glycosylated asparagines of the same proteins [24]. This suggests that the variations that lead to changes of the glycosylation pattern of a protein can be damaging. To better understand if there is a bias in terms of variations that affect the N-glycosylation motif, we calculated the frequency of all three amino acids that are affected by variation (Table 3). The results indicate that there is a slightly lower percentage of variation in asparagine at the N-glycosylation motif compared to asparagines that lie outside of the N-glycosylation motif (this holds true for serine and threonine) (Table 3).

### Functional Analysis

To better understand the distribution of the variants in functional protein groups, we have analyzed our dataset to see if there is over- or under- representation (compared to the human proteome) of functional elements such as domains and pathways. We find that the most significantly over-represented Pfam domains (out of a total of 12,015 Pfam domains), both in the loss and gain datasets, are the Immunoglobulin I-set domain (p-value 3.84E-25), Tyrosine kinase (p-value 5.20E-24), Fibronectin type III domain (p-value 2.90E-23), and regular and calcium-binding EGF domain (p-value 4.06E-21). All of these domains are found in secreted proteins and/or on the extracellular part of membrane proteins. The domain analysis shows an over-representation of proteins that are involved in cell-adhesion, cellular proliferation and wound healing. This theme is repeated in all of the functional analyses described below.

#### Ingenuity Pathway Analysis

Ingenuity Pathway Analysis (IPA) was used to further explore functional aspects of our protein dataset. We describe here the top 3 significant results (the remaining categories are described in Table S4). A total of 134 N-glycoproteins in our dataset are involved in cell-to-cell signaling and interaction (CTCSI) and 122 are involved in cellular movement (CM), the top two molecular and cellular functions (Figure 2). CTCSI represents primarily proteins involved in adhesion and activation of various cells of the immune system. Specific enriched immune functions include responses of phagocytes, neutrophils and antigen presenting cells. The main proteins that contribute to the immune responses were found to be transmembrane receptors such as toll-like receptor 4 (TLR4), beta 2 microglobulin (B2M), a component of MHC class 1 molecule, Fas (TNF receptor superfamily, member 6), IL2RA (interleukin 2 receptor alpha) and others. There are several reasons why these proteins could be more affected by polymorphism in the N-glycosylation sites. For example, it is known that N-linked glycosylation of toll-like receptors is required for their translocation to the cell surface [42]. N-linked glycosylation is known to activate NFkappaB signaling, an important pathway in the immune response [43].

In the cellular movement category, migration, invasion and movement of cancerous cells are the major enriched classes. Most of the proteins contributing to this important feature are tyrosine kinases such as PTK2B, ERBB3, EPHB4, and FGFR1. Tyrosine kinases play a vital role in many cellular functions such as cell differentiation, proliferation, motility and cell survival. Mutations in these kinases lead to deregulation of various cellular processes resulting in unbalanced proliferation and differentiation, which can result in cancerous growths [44]. Receptor tyrosine kinases have been suggested as drug targets in cancer therapy but cancer cells tend to develop resistance to these therapeutics. Targeting N-linked glycosylation, a highly regulated step for maturation of these receptors, is a novel therapeutic idea. A recent study pointed out that inhibiting N-linked glycosylation reduces receptor tyrosine kinase signaling and radiosensitizes tumor cells [45]. The authors also suggested that enzymatic steps regulating N-linked glycosylation can serve as novel targets in the treatment of malignancy in general. The next top functional category predicted by IPA was cellular function and maintenance. Most glycosylated proteins in this category are involved in cytoskeleton organization such as organization of actin filament extension of cellular protrusions; transportation through ion-channels (e.g. flux of calcium ions) and depolarization of cellular and mitochondrial membranes.

Analysis of physiological system function and development identified over-representation of proteins involved in tissue, organismal and, hematological system development (Figure 2). The tissue development category comprises development of various organs such as liver, kidney, and bone by adhesion of cells. The organismal development refers to a broad category comprising developmental stages from zygote to multicellular animal or from young adult to aged adult. All the major processes in this category are influenced by polymorphic N-glycoproteins. It is not surprising to note such a global effect of N-linked glycosylation because this pathway expanded significantly in multicellular organisms especially in the vertebrates. It has been shown that N-glycosylation influences many biological processes in an organ and species specific manner [46]. Many of the knockout mouse models targeting enzymes of the N- glycosylation enzymatic pathway are embryonic lethal since they affect organ development.

Pathway analysis by IPA identified 13 of 35 glycoproteins involved in the coagulation system among the polymorphic glycoproteins (p<0.05). Similarly, 11 out of 29 molecules in the intrinsic prothrombin activation pathway matched our dataset (p<0.05). The top two pathways, the coagulation system (extrinsic) and the intrinsic prothrombin activation pathway, refer to the coagulation cascade [47] where glycosylation is known to be important [48].

#### Clustering analysis

To further understand protein families, we have clustered all the proteins based on significant end-to-end similarity. Major clusters include the G protein-coupled receptor (GPCR) cluster (42 proteins), the kinase (EGF receptor subfamily) cluster (27 proteins), the HLA class I histocompatibility antigen type proteins (17 proteins) and proteases (13 proteins) (see Table S5). This is in agreement with the results of the domain and pathway analyses.

With 42 members (expected 38; hence not over-represented) GPCRs form the largest cluster in our dataset. Even though the GPCRs are not over-represented, we provide a brief description of the cluster members as this cluster is the largest in our dataset. All the proteins in this group are membrane proteins with 20 olfactory receptors representing a major subset. It is known that glycosylation of GPCRs, the largest family of cell-surface receptors, is central to normal reproduction [49], [50]. Glycosylation of these receptors also affects stability and localization of the proteins. For example, mutation of glycosylation sites of the glucagon-like peptide 1 receptor (GLP1R) result in aberrant distribution with most of the receptor localized in the endoplasmic reticulum and golgi compartments instead of the cell surface [29]. This is one of the reasons why we include all the proteins in our analysis irrespective of their annotated location in UniProtKB/Swiss-Prot.

The kinase (EGF receptor subfamily) cluster is over-represented for the tyrosine kinases (expected 6, observed 21) including receptor tyrosine-protein kinase erbB-4 (ERBB4), fibroblast growth factor receptor 2 (FGFR2) and tyrosine-protein kinase Tec (TEC). All of the proteins in this cluster are ATP-binding proteins and the majority of them (21 out of 27) are known membrane/secreted proteins. 17 of the 21 tyrosine kinases in our list are annotated as receptors with a ligand-binding extracellular domain and a catalytic intracellular kinase domain. Analysis of this subset showed that 15 proteins contain a new NXS/T in the extracellular region and are expected to be functionally affected by N-glycosylation [24], [44]. We further explored the connection of these proteins with diseases and, as expected, several map to cancer diseases, with the majority implicated in colorectal and breast cancers. For example, the variat T140I in ERBB4, leads to loss of a glycosylation motif and altered development of colorectal adenocarcinoma [51].

HLA class I histocompatibility antigens (17 proteins) consist of membrane/secreted proteins including HLA class I histocompatibility antigen, beta-2-microglobulin, and T-cell surface glycoprotein CD1c. The majority of the proteins are involved in immune responses as part of the MHC protein complex. This group of proteins is also over-represented (expected 7, observed 17) in our dataset. It is known that glycosylation plays an important role in molecules of the MHC that present antigens to T cells [52] as part of the adaptive immune response [53].

Serine proteases represent another set of proteins with over-represented NXS/T variants (expected 7, observed 13). N-glycosylation of the proteases and their substrates plays an important role in protease activity. The serine proteases participate in the processes of blood coagulation, apoptosis and inflammation [54]. All of the processes described above represent a recurrent theme in our analysis and point out the overall importance of N-glycosylation in these pathophysiological pathways. Overall the protein families that are over-represented in our dataset based on cluster analysis show that protein involved in immunity, blood coagulation and kinase activity are the major types that undergo changes in glycosylation. A survey of nsSNV in these proteins reveals that there is an abundance of nsSNVs in the blood coagulation pathway proteins and serine proteases (number of amino acids in the human proteome 11293923, total nsSNVs 494983; number of nsSNVs inside data set 1959, expected 1335, p-value 0.05). The difference between the expected and observed is less for HLA class I histocompatibility antigens and related proteins with the MHC domain (number of nsSNVs inside data set 1952, expected 2070, p-value 0.40) and for the kinases with the protein tyrosine kinase domain (number of nsSNVs inside data set 5670, expected 4749, p-value 0.09). It is possible that the blood coagulation group of proteins indeed has a high level of variation or there is a distinct bias in the research community towards these groups of proteins. Availability of more data will allow us to identify if there is indeed a bias in the research focus, which subsequently results in more depositions of SNVs for some of these functional groups.

In some rare cases, such as HLA class I histocompatibility antigens (HLA-B and HLA-C) and tubulins (TBA1C_HUMAN and TBA1B_HUMAN), the change that leads to gain of glycosylation occurs in the same N-glycosylation motif in the related proteins and could indicate a common functional effect in the gene family. Unlike the HLA class I histocompatibility antigens, which are membrane proteins, the tubulins are found in the cytoplasm and cytoskeleton and therefore are not expected to be glycosylated. There are reports on tubulin glycosylation and it has been suggested that some portions of the α and β tubulin are translated in the endoplasmic reticulum and hence it is possible they might be glycosylated [55], [56].

#### Structural analysis

We performed structural analysis to elucidate where the modifications are located. We found that 277 out of 1091 proteins have structures available in the Protein Data Bank (PDB) [57] at the position of variation. It was previously believed that the glycosylated region has to adopt beta-turn conformation [58]. Later it was found that many turns or loop conformations are suitable to produce an exposed glycosylation site [59]. Petrescu et al. analyzed the secondary structures at the N-glycosylation sites and found that turns and bends are favored with ∼27% and 18% of occupied sites on turns and bends with the lowest glycosylation site incidence (11%) on helices [60]. We notice that the majority of the variations are within the loop and bend regions (32% are in loop regions, 16% in bends, 10% in the strands and 22% in the helices). Zielinska et al. predicted the secondary structure localizations in mouse glycoproteins and suggested that 71% of the N-linked asparagines are located in the loop regions. [9]. A survey of the distribution of SNPs in the different secondary structures shows that there is no bias in the occurrence of nsSNPs in the different secondary structural elements (number of SNPs inside loops/turns: 1385, expected 1305; strands: 9374 expected: 8355; helix: 14118, expected: 13376). These findings indicate that although the nsSNPs that affect N-glycosylation motifs are enriched in the loop and bend region (48%) and under-represented in the other secondary structural elements, the enrichment is not as high as expected based on the fact that 71% of the aspargines are located on loops. This can be attributed to the fact that N-glycosylation sites in loops are functionally important and hence, less likely to contain variation.

We found that 189 proteins in our dataset are secreted based on UniProtKB/Swiss-prot annotation and prediction results from WoLF PSORT [61]. Network analysis followed by Gene Ontology enrichment analysis of these 189 proteins using STRING [62] reveals that a large number of these proteins are involved in blood coagulation. Response to wounding, platelet activation, blood coagulation and hemostasis belong to the top terms, ranked based on p-value. We also saw blood coagulation pathway as one of the top canonical pathways predicted by IPA and over-representation of this set in our clustering and domain analysis was also observed. Antithrombin III, a protein which loses an NXS/T site due the N167T substitution, is found to interact with ten proteins of which 9 are involved in blood coagulation pathway based on STRING network analysis (Figure 3). We have selected this protein to carry out structural and functional analysis as described below. Availability of structure in PDB (2ANT) of antithrombin III allowed us to model the variation and see the effect of loss of glycosylation motif (UniProtKB accession P01008, position 167NKS169; PDB id 2ANT, position 135NKS137). As there is no carbohydrate attached to the glycosylation motif 167NKS169, this is an example of changes can be observed at the structural level due the loss of the glycosylation motif. Modeling results show that the loss of glycosylation motif does not result in significant changes to the structure when compared with 2ANT (0.42Å RMSD). Our model also shows that the variation increases the relative solvent accessibility (RSA) of that region from 0.69 to 1.00, which could have an effect on heparin binding (heparin binding site: F156– G180 [63]). Bayston et al. [64] investigated the role of loss of glycosylation on antithrombin deficiency in an asymptomatic individual (and family) with borderline levels of antithrombin. Direct sequencing confirmed the mutation that leads to N167T substitution and loss of glycosylation near the heparin interaction site which results in deficiency of total antithrombin in the plasma of the asymptomatic individual and her family. The author’s hypothesize that the loss of glycosylation results in more conformational flexibility which in turn results in lower affinity binding to heparin. Our modeling studies do not show any conformational flexibility but it is possible that the carbohydrate assists in heparin binding and its loss could lead to lower binding affinity to heparin.

In the second example we chose a protein that has a carbohydrate attached to the glycosylation motif. Based on our functional analysis using IPA, we had observed that cathepsin D, an acid protease, is in the list of top cellular, molecular functions and diseases such as metabolic, genetic and developmental disorders and has a structure available with a carbohydrate attached at the variant glycosylation sequon. The loss of the N-glycosylation motif is caused by the variation T136A in this protein (UniProtKB accession P07339, position: 134NGT136; PDB id 1LYA, position: 70NGT72). While there were no major structural changes, we did observe a loss of a hydrogen bond that originally existed between the threonine residue and the sugar (Figure 4a and 4b). This also changed the charge distribution at the site making it more neutral with the alanine present. Also, there was an increase in solvent accessibility of the asparagine from 0.45 to 0.62 and a decrease in the accessibility at the site of the variation from 0.53 for threonine to 0.45 for alanine. There is evidence in the literature for the role of glycosylation in the regulation of cathepsin D in cancers [65]. Cathepsin D contains two N-glycosylation sites at positions 134 and 263 and both sites are important for targeting to lysosomes [66]. While we cannot test this with our data, it is tempting to speculate that the occurrence of the nsSNV in one of the sites interferes with its stability and lysosomal targeting. The example demonstrates the potential of such analysis to stimulate laboratory verification of these interesting functional characteristics.

To summarize, the results obtained from this study provide for the first time, not only a comprehensive survey of the effect of nsSNVs on the human glycoproteome, but also a framework that will assist glycobiologists with sorting and ranking of the datasets available in Tables S1, S2, S3, S4, and S5 to identify proteins of interest. These candidates can be further analyzed in the laboratory to test functional predictions. The structural analysis of antithrombin-III and cathepsin D provides a method that can be applied to study the functional implications of variation that lead to change of glycosylation in other proteins. The requirement is that these proteins either have a solved 3D structure or have closely related proteins with solved structures. The amount of information available on the 1091 proteins described in this paper is vast and is constantly being updated by the UniProtKB/Swiss-Prot curation team. A brief description is provided here to facilitate browsing the datasets in UniProt. Copy accessions from Tables S1, S2, S3, S4, and S5, paste them in the UniProt identifiers input box at http://www.uniprot.org/batch and click on retrieve; in the intermediate window click on UniProtKB to view the results in tabular format; next, one can either browse individual proteins by clicking on the accession numbers or browse by Gene Ontology or UniProtKB/Swiss-Prot keywords by clicking. Availability of more SNV data from ongoing genome sequencing projects, validation of N-glycosylation sites, and availability of more functional data on individual proteins will undoubtedly unravel how variation in this important post-translation modification affects human biology.

## Supporting Information

Table S1 **N-linked glycosylation loss and gain sets.**
(XLSX)

Table S2 **Distribution of N-linked glycosylation loss and gain sites in proteins.**
(XLSX)

Table S3 **Endoplasmic reticulum signal peptide prediction results.**
(XLSX)

Table S4 **Ingenuity Pathway Analysis (IPA) results.**
(XLSX)

Table S5 **Clustering analysis results.**
(XLSX)
